# Uncovering the mystery of ferroelectricity in zero dimensional nanoparticles

**DOI:** 10.1039/c8na00131f

**Published:** 2018-11-01

**Authors:** Yury A. Barnakov, Ighodalo U. Idehenre, Sergey A. Basun, Trevor A. Tyson, Dean R. Evans

**Affiliations:** Air Force Research Laboratory, Materials and Manufacturing Directorate Wright-Patterson Air Force Base Ohio 45433 USA dean.evans@us.af.mil; Azimuth Corporation 4027 Colonel Glenn Highway, Suite 230 Beavercreek Ohio 45431 USA; University of Dayton, Department of Electro-Optics and Photonics Dayton Ohio 45469 USA; Department of Physics, New Jersey Institute of Technology Newark NJ 07102 USA

## Abstract

It is generally accepted that chemically synthesized nanoparticles lose their ferroelectricity (spontaneous polarization) as the particles become smaller. In contrast, ball-milled ferroelectric nanoparticles have an enhanced ferroelectric response at remarkably small sizes (≤10 nm). Although prior theory suggests that surface stress influences ferroelectricity, the source of such a stress and how it physically influences ferroelectricity in zero-dimensional nanoparticles has remained a mystery. In this paper, we demonstrate that the top-down approach of wet ball-milling not only results in fragmented materials on the nanoscale, but it also is responsible for a mechanochemical synthesis of metal carboxylates forming at the nanoparticles' surface. We prove that the presence of such a compound with a particular type of binding mode chemisorbed at the nanoparticles' surface is responsible for producing surface stress. This surface stress results in a stabilization and dramatic enhancement of the spontaneous polarization, which is 5 times greater than that of the bulk material and 650 times greater than what is measured in materials fabricated using standard chemical synthesis techniques. The results of this study have further led to the development of a new process that produces ferroelectric nanoparticles (≤10 nm) with uniform shape and size using a combination of wet chemistry and mechanochemical synthesis.

## Abstract 2

A crystalline organic shell provides a 650 times increase in spontaneous polarization in nanoscale BaTiO_3_ core–shell superparticles.

## Graphical abstract

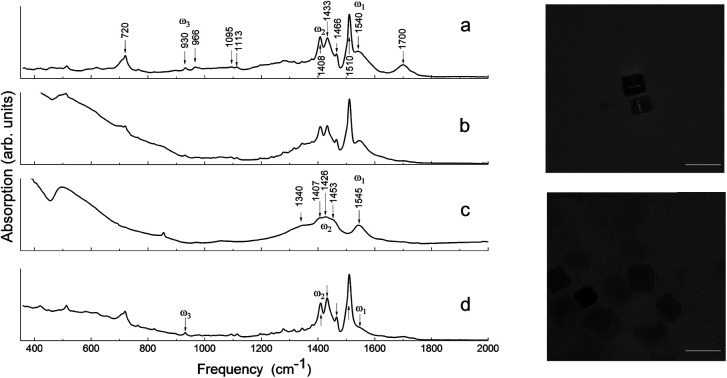

## 1. Introduction

It has been well-documented in the literature that the spontaneous polarization/dipole moment dramatically reduces in magnitude as the particle/grain size of conventional ferroelectrics becomes less than 100 nm.^1^ However, there have been several papers on the discovery of ferroelectricity in BaTiO_3_ nanocolloids/nanoparticles prepared by wet ball-milling in nonpolar heptane with oleic acid. These samples demonstrate very large values of spontaneous polarization;^2,3^ this is in strong contradiction to what is observed in nanoparticles/nanograins produced *via* traditional wet chemistry routes and ceramic fabrication, *i.e.* solvothermal synthesis, sol–gel, spark plasma sintering, *etc.*^1,4,5^ Although the physics of ball-milled ferroelectric nanoparticles has not been well understood, they have been widely used in liquid crystal studies over the past couple of decades showing remarkable enhancements of both electrical- and electro-optical responses.^6–9^ Moreover, ball-milled ferroelectric nanoparticles have been used outside of optical/liquid crystal applications for molecular catalysis applications, where the large dipole field from the ferroelectric nanoparticles replaces the need for any external bias.^10^

Prior to this current paper, ball-milling was mainly considered as a simple top-down approach for fabricating *randomly shaped* ferroelectric nanoparticles, without a formal explanation for the existence of ferroelectricity on such a small scale. Since any observed ferroelectricity was extremely weak at best in chemically produced nanoparticles, opposed to the strong response detected in ball-milled nanoparticles, it was assumed that surface stress was mechanically-induced during the high energy ball-milling process.^2,11,12^ Although theory in 2007 and experimental evidence in 2010 supported this claim,^2,11,12^ it was only supposition that the high impact velocity of the milling beads could impart a surface stress in the particles during grinding. The exact mechanism for the creation of the stress that provided such a large spontaneous polarization was unknown. It was also believed that the oleic acid, a typical choice of surfactants, served solely as a means of preventing particle aggregation.^9,12–14^ However, a recent discovery in ref. 15 shows that ball-milling not only results in a top-down approach for the mechanical creation of ferroelectric nanoparticles, it also results in a chemical reaction at the nanoparticle surface (*i.e.* a mechanochemical formation of large quantities of barium oleate from the oleic acid starting material). With the conclusions drawn in ref. 15, a revision of this common point of view, that ball-milling only results in the fragmentation of the particles, is required.

In this paper, it is shown that a metal carboxylate bulk compound, resulting from mechanochemical synthesis, plays a key role in stabilizing/enhancing ferroelectricity in wet ball-milled nanoparticles. A relationship is established between the spontaneous polarization of these nanoparticles and their surface interactions with surrounding molecular entities. Evidence is presented proving that only a certain type of coordination binding mode of carboxylates possessing a molecular packing order/crystalline structure is chemisorbed at the nanoparticles' surface; this particular metal carboxylate is responsible for a surface stress and in turn a stabilization and dramatic enhancement of ferroelectricity (spontaneous polarization). The identification and explanation of the physical differences of the carboxylate functional groups and the types of coordination binding modes for both chemically (traditional wet chemistry approaches) and mechanically (ball-milling) produced nanoparticles is also investigated. Furthermore, a new technique/process is presented that overcomes the major drawback of ball-milled nanoparticles, *i.e.* their random shape. This new technique results in a controllable uniform shape and size of highly ferroelectric nanoparticles, whose properties are defined by the type of coordination binding mode of a barium oleate COO^−^ carboxylate. The difference in the binding modes is what allows for the typical amorphous (quasi-bridging bidentate structure) organic coating to become a crystalline (chelate bidentate structure) “shell” as a result of mechanochemical synthesis. Finally, the physical mechanism enabling a surface stress and thus a ferroelectric stabilization and enhancement is determined for the first time. The surface stress, which is shown in this work to be an epitaxial-like stress/strain between the inorganic core and the surrounding crystalline organic component, is responsible for a 650 times (>130 μC cm^−2^) enhancement of the spontaneous polarization in BaTiO_3_ nanoparticles compared to non-stressed BaTiO_3_ nanoparticles; this is a 5 times increase compared to the value found in bulk BaTiO_3_ (26 μC cm^−2^). The fundamental understanding of the surface physics and ability of its manipulation opens many doors for producing surface relevant properties for various applications, *e.g.* catalysis, liquid crystal displays, photorefractive beam coupling in hybrid devices, *etc.*^6–10,16^ This discovery paves the road toward the creation and control of ferroelectricity in uniformly-shaped “zero dimensional” ferroelectric “superparticles”, where the term superparticles (used in ref. 2) applies to this new class of nanoparticles where the spontaneous polarization belongs to the inorganic–organic hybrid core–shell system (core nanoparticle and functional shell) and not just the inorganic nanoparticle itself.

## 2. Background

It is widely accepted that ferroelectric materials undergo a degradation of their hallmark properties with size reduction, and even a complete disappearance at a critical size.^1,17–19^ This size dependence is manifested in the reduction of the Curie temperature, the diffusive character of the phase transition, and the suppression of the spontaneous polarization. Therefore, the current challenge in nanoscale ferroelectrics is to stabilize and enhance ferroelectricity, while reducing the materials' dimensionality. Depending on the dimensionality of the nanomaterial, either 2-D thin films or 0-D nanoparticles, different strategies and mechanisms of ferroelectricity enhancement may be employed. Thin 2-D film technologies have demonstrated a steady and tangible success with well-developed fabrication procedures and a deep understanding of the materials' physics. As such, Choi *et al.* employed an elastic strain engineering concept based on a lattice mismatch, achieving a 230% (60 μC cm^−2^) enhancement of the spontaneous polarization (compared to bulk BaTiO_3_, 26 μC cm^−2^) in epitaxially grown BaTiO_3_ multilayered thin films;^20^ this is supported by Ederer *et al.* whose theory shows a linear dependence of the spontaneous polarization as a function of elastic strain.^21^

A different scenario is considered for 0-D nanoparticles synthesized *via* conventional wet chemistry routes. The relaxing synthetic conditions set modalities for the growth of nanoparticles with paraelectric centrosymmetric structures, where the properties are mainly determined by the inorganic core of the nanoparticle with no functionality from a coating/shell. Numerous experimental works fall into this paradigm supported by the size dependent phenomenological Landau–Devonshire–Ginzburg (LDG) theory.^22,23^ For instance, Caruntu *et al.* synthesized and characterized well-defined single domain BaTiO_3_ nanocubes and nanospheres.^4,24^ The value of spontaneous polarization of similar samples as found in ref. 4 (provided to us by G. Caruntu and measured in our laboratory) was 0.2 μC cm^−2^ (about 1% the value for the bulk crystal); the fact that these nanoparticles existed in the ferroelectric phase was an improvement to those that had been confined to the paraelectric phase.

Alternatively, a top-down approach (ball-milling) has been used to fabricate small BaTiO_3_ nanoparticles (randomly-shaped),^2,3,7–10,12–15,25^ which has revolutionized the field by offering a simple and effective technique to create highly ferroelectric BaTiO_3_ nanoparticles. Morozovska *et al.* proposed a mechanism for such an enhancement to be linked to surface stress/strain;^11^ direct experimental proof of the presence of such a surface stress in randomly-shaped ball-milled BaTiO_3_ nanoparticles with large spontaneous polarization values (100–120 μC cm^−2^) is demonstrated in ref. 2, 3 and 12, although the source of the stress remained a mystery.

To further improve the ferroelectric properties and overcome these fundamental limitations, it is necessary to explore novel mechanisms of enhancement by taking into consideration not only the nanoparticle core, but also its surface and interface. This current work identifies and describes the source of surface stress, thus unraveling the mystery of the strong ferroelectric effect on a nanoscale (≤10 nm); it also investigates functional surface bonds, unachievable by conventional chemistry,^26,27^ which provide the required conditions for the enhancement of ferroelectric properties.

## 3. Experimental

### 3.1. Synthesis of BaTiO_3_ nanocolloids and their components

A series of materials were prepared using various methods: (1) ball-milled BaTiO_3_ nanoparticles were prepared using commercial Aldrich powder as reported in ref. 2, 8, 9, 12, 13 and 15, (2) BaTiO_3_ nanocubes (pre-milled) were created using solvothermal synthesis following the method used in ref. 4, and (3) post-milled BaTiO_3_ nanocubes were prepared by ball-milling the nanocubes obtained in (2) using a planetary high energy ball-mill Retsch PM 100. For this latter case, a slurry comprising 0.1 g of synthesized BaTiO_3_ nanocubes, 0.1 g of oleic acid and 15 ml of heptane was immersed into a ball-mill crucible filled with 2 mm ZrO_2_ beads and subjected to rotation at 500 rpm for 5 hours; the produced nanoparticles/nanocubes were converted to powder using anhydrous ethanol with sequential washing/drying at ambient temperature, resulting in a white precipitate. All starting materials included oleic acid unless noted otherwise. Note: the use of the term nanocubes refers to nanoparticles with a controlled cubic shape achieved using the solvothermal synthesis technique. The same milling conditions were also used for the creation of ball-milled BaTiO_3_ nanoparticles of random shape in (1) using a precursor BaTiO_3_ powder supplied by Aldrich. Individual components of ball-milled commercial barium oleate (Pfaltz & Bower), and unmilled commercial BaTiO_3_ (Aldrich) powder were also investigated.

### 3.2. Characterization of BaTiO_3_ nanoparticles

Optical, electrical, and morphological studies were performed on the above-mentioned nanomaterials. Temperature dependent infrared (IR) absorption spectra were measured using Fourier-transform infrared spectroscopy with an FTIR-NIR Bruker spectrophotometer in an Attenuated Total Reflection (ATR) configuration over the range of 400–3000 cm^−1^ with 2 cm^−1^ resolution. AC displacement current density measurements, as described in ref. 2, 3 and 10, provided values of the spontaneous polarization of samples that were diluted (1 : 1000) with heptane containing 0.4% by weight of oleic acid. Surface morphology was investigated using a Philips CM 200 transmission electron microscope with 200 kV accelerating voltage. In addition, X-ray diffraction (XRD) measurements were conducted on dried out solutions of samples ball-milled in heptane with oleic acid (weight ratio of oleic acid : BaTiO_3_ was 1 : 1) and without oleic acid, as well as ball-milled commercial barium oleate and commercial BaTiO_3_ starting powders. Thermogravimetric analysis (TGA) measurements were conducted to verify the organic phase of the core–shell nanoparticles using a TA Instruments Q500 TGA.

## 4. Results and discussion

To explore the effects responsible for the ferroelectric stabilization and enhancement and provide empirical evidence to support these claims, several samples were studied. These samples are referred to throughout the text as: “ball-milled nanoparticles” (using commercial starting materials, Aldrich powder), “pre-milled nanocubes” (produced by solvothermal synthesis following the recipe from ref. 4), “post-milled nanocubes” (solvothermal synthesized nanocubes subjected to post-growth high-energy ball-milling as a means of mechanochemical synthesis), and “ball-milled commercial barium oleate”.

**Fig. 1:**
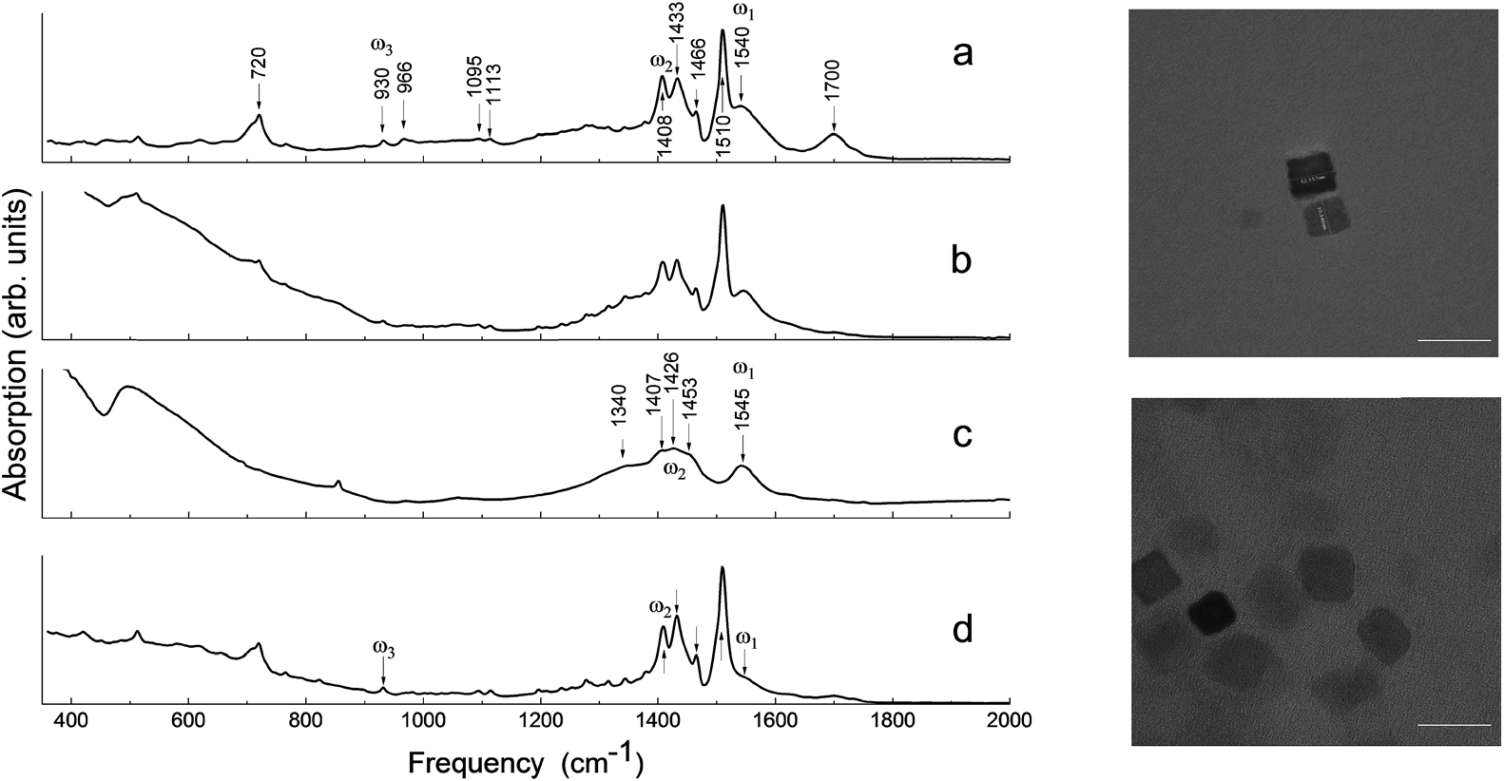
(Left) FTIR absorption spectra of (a) ball-milled commercial barium oleate, (b) ball-milled BaTiO_3_ (Aldrich) nanoparticles, (c) pre-milled BaTiO_3_ nanocubes, and (d) post-milled BaTiO_3_ nanocubes. *ω*_1_ corresponds to the asymmetrical stretching modes and *ω*_2_ corresponds to the symmetrical stretching modes. *ω*_3_ corresponds to the deformation mode of COO^−^. (Right) TEM images of synthesized BaTiO_3_ nanoparticles: pre-milled (top) and post-milled (bottom). The white bar depicts a 20 nm scale.

The identification of both the local structure of barium oleate and the long-range molecular ordering can be achieved using FTIR spectroscopy.^28^Fig. 1 shows the FTIR absorption spectra for: ball-milled commercial barium oleate, ball-milled BaTiO_3_ nanoparticles, pre-milled BaTiO_3_ nanocubes, and post-milled BaTiO_3_ nanocubes. Note, the average nanoparticle size reduces as the milling time increases, asymptotically reaching a minimum average size of approximately 9–10 nm.^9,13^ For this reason ball-milling of the solvothermal synthesized ∼10 nm nanocubes was not expected to reduce the size any further, although it was expected that it could affect the chemical properties of the organic component (coating/shell) and the level of surface stress. Note: the surrounding organic material coating the nanoparticle will be referred to as a “shell” for the remainder of this paper. TEM images of synthesized BaTiO_3_ nanocubes are shown in Fig. 1 for both the pre-milled and the post-milled material. There is no significant change in the average size or shape of the pre- and post-milled nanocubes; this is a dramatic improvement in uniformity over the top-down approach of ball-milling bulk material (see ref. 13 and 14), which did not allow for any control of the shape of the nanoparticles.

**Fig. 2:**
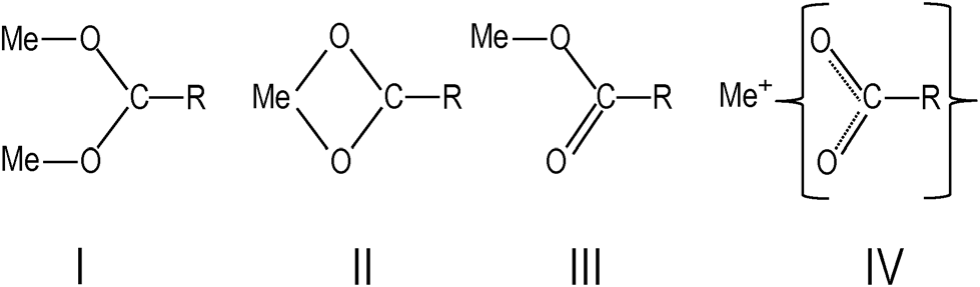
Types of carboxylate coordination binding modes: (I) bridging bidentate, (II) chelate bidentate, (III) covalent monodentate, and (IV) ionic monodentate,^28–31^ where R is C_17_H_33_.

**Fig. 3:**
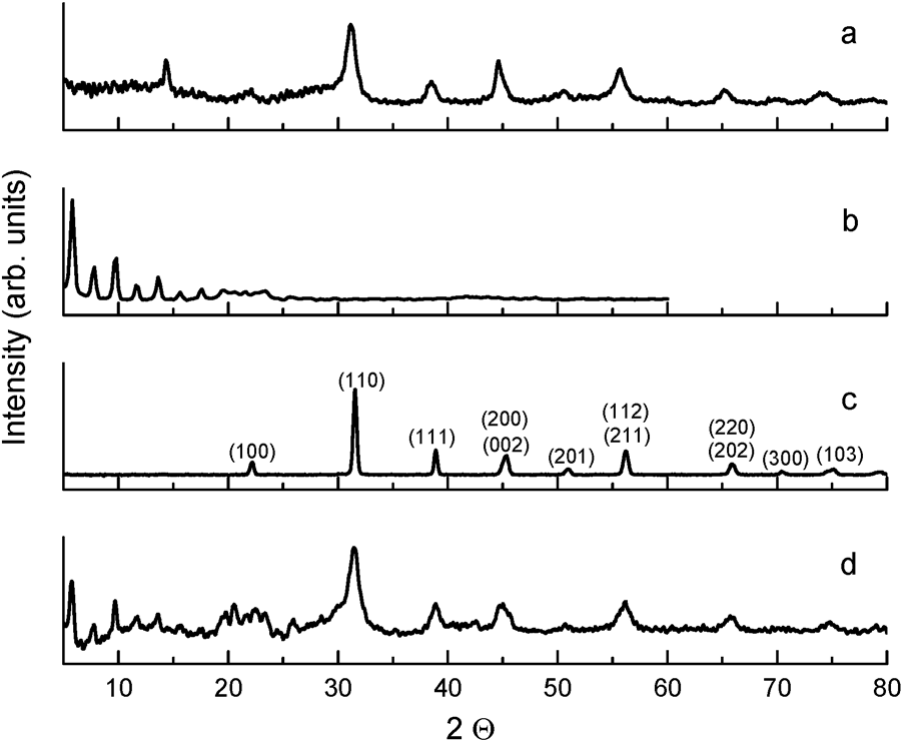
XRD patterns of: (a) 25 h ball-milled BaTiO_3_ without oleic acid in heptane, (b) ball-milled commercial barium oleate, (c) commercial BaTiO_3_ (Aldrich, 130 nm) powder/starting material, and (d) 25 h ball-milled BaTiO_3_ with oleic acid in heptane.

In Fig. 1, the FTIR absorption spectra of (a), (b) and (d) are nearly identical with only minor differences, such as the feature centered around 1710 cm^−1^ and the low-frequency tail. The 1710 cm^−1^ absorption band is a “fingerprint” of the vibrational mode of carbonyl (C=O), which is associated with the presence of free oleic acid molecules found in commercial barium oleate, and the broad low-frequency tail is due to the BaTiO_3_ absorption. The spectrum of the pre-milled BaTiO_3_ nanocubes (Fig. 1c) is distinctly different from the others suggesting a different type of COO^−^ coordination binding mode. It is known for different types of carboxylates, that the splitting in energy between asymmetrical and symmetrical stretching vibrations (*ω*_asym_ − *ω*_sym_ = *Δ*) is indicative of the type of carboxylate coordination binding mode.^29,30^ Based on this criteria, the four major carboxylate structures can be characterized as follows (see Fig. 2):^30,31^ (I) *Δ* > 110 cm^−1^ – a bridging bidentate, in which each oxygen of the carboxylate covalently bonds to a different metal cation; (II) *Δ* < 110 cm^−1^ – a chelating bidentate, in which charge of the carboxylate is shared across the oxygen atoms, and both form covalent bonds with the metal cation; (III) *Δ* > 200 cm^−1^ – a covalent monodentate, in which a negatively charged oxygen of the carboxylate forms a covalent bond with the metal ion; and (IV) an ionic monodentate, in which one negatively charged oxygen of the carboxylate and a metal cation form an ionic bond. Typically, the characteristic IR absorption bands for metal carboxylates, in particularly the carboxylate group (COO^−^), are in the range 1500–1610 cm^−1^ for asymmetrical stretching modes (*ω*_1_ in Fig. 1) and 1300–1450 cm^−1^ for symmetrical stretching modes (*ω*_2_ in Fig. 1). For the samples shown in Fig. 1a, b and d, the sharp absorption band at 1510 cm^−1^ and the shoulder around 1540 cm^−1^ are related to asymmetrical stretching modes, while the 1408 cm^−1^ band is related to the symmetrical stretching modes. The 1466 cm^−1^ band is assigned as a CH_2_ bend (*δ* (CH_2_) scissoring band),^29^ however, an assignment of 1433 cm^−1^ is not straightforward. It may be related to symmetrical stretching such that there are two pairs of *ω*_asym_ and *ω*_sym_ frequencies (*ω*_asym_ = 1510 cm^−1^, *ω*_sym_ = 1408 cm^−1^ and *ω*_asym_ = 1540 cm^−1^, *ω*_sym_ = 1433 cm^−1^) with a value of *Δ* for both cases being <110 cm^−1^, justifying the chelate structure (Fig. 2, type II). On the other hand, the 1433 cm^−1^ feature may be related to CH_2_ vibrations resulting from the overlap of *ω*_1_ (COO^−^) stretching and *δ* (CH_2_) scissoring bands. In this case, a mixture of both chelate and quasi-bridging (*Δ* value for the pair 1540 cm^−1^ and 1408 cm^−1^ is 132 cm^−1^) coordination binding modes would need to be considered. Nevertheless, the presence of a chelate structure is dominating in these samples (for example, see the 1510 cm^−1^ strong absorption in the cases of Fig. 1a, b and d); this type of local structure favors an ordered arrangement of barium oleate molecules at the nanoparticle surface. Equally spaced absorption bands in the range 1180–1350 cm^−1^ (progressive bands – attributed to wagging/twisting vibrations of chains of successive methylene groups of the barium oleate molecule) and the 720 cm^−1^ feature (attributed to rocking vibrations of chains of successive methylene groups of the barium oleate molecule) both serve as proof of crystallization of barium oleate.^29–31^ This claim is supported by the work of *Koga* and *Matuura*,^32^ who extensively studied the relationship between FTIR absorption spectra and the structure of metal carboxylates. They showed that the type of carboxylate coordination can dictate the character of the molecular packing order, ranging from crystalline basalt to amorphous structures, which is the case for BaTiO_3_ nanoparticles/nanocubes with oleate shells in chelate and quasi-bridging configurations, respectively. Further evidence of the crystalline structure of the chelate bidentate in the ball-milled samples is shown in the diffraction patterns of the XRD data found in Fig. 3.

The ball-milled sample with oleic acid presented in Fig. 3d is comprised of two phases: an organic phase of barium oleate (*i.e.* mechanochemical synthesis converted oleic acid to an ordered chelate-structured metal carboxylate, Fig. 2, type II) characterized by the equally spaced sharp diffraction peaks below 20° of 2*θ*, and an inorganic phase of BaTiO_3_ nanoparticles characterized by broad diffraction peaks above 20° of 2*θ*. Individual milled organic and unmilled inorganic components are shown in Fig. 3b and c, respectively. Thermogravimetric analysis (TGA) measurements confirmed the presence of an organic phase in the sample and estimated its quantity to be at least 50% by weight, which is in agreement with the results using an independent method in ref. 15. It is worth noting that the sharp diffraction peaks below 20° of 2*θ* in Fig. 3b and d demonstrate the crystalline nature of the organic component found in pure (ball-milled) barium oleate in an ordered chelate coordination binding mode and ball-milled BaTiO_3_ (with oleic acid), respectively. It is worth emphasizing that two independent methods, FTIR and XRD, both demonstrate that milled nanoparticles/nanocubes have crystalline organic shells, as opposed to the case of unmilled/pre-milled materials. For the case of the ball-milled BaTiO_3_ without oleic acid in heptane, diffraction peaks from BaTiO_3_ and barium oleate are not observed below 20° of 2*θ* (Fig. 3a). The diffraction peak at ∼14° seen in Fig. 3a is not present in Fig. 3d. It is also worth noting that the absorption spectra of the samples used for Fig. 3a and c (no oleic acid in the starting material) do not resemble the spectra of the BaTiO_3_ samples milled with oleic acid (data not shown). The sample used for Fig. 3d gives a very large spontaneous polarization (due to the presence of the metal carboxylate), unlike the sample used for Fig. 3a (which contains no metal carboxylates). We therefore conclude that the source of this feature at ∼14° does not play a role in the spontaneous polarization enhancement. The source responsible for this diffraction peak may have been formed as a byproduct of ball-milling BaTiO_3_ in heptane (without oleic acid).

In contrast to the discussion above, the FTIR absorption spectrum of the pre-milled nanocubes in Fig. 1c shows broad asymmetrical stretching (centered at 1545 cm^−1^ with no sign of the 1510 cm^−1^ absorption band) and broad symmetrical stretching (centered at 1426 cm^−1^ including the 1408 cm^−1^ band) carboxylate modes with an energy splitting (*Δ*) of either 119 cm^−1^ or 137 cm^−1^; in both cases the *Δ*-values correspond to the quasi-bridging binding mode (Fig. 2, type I). This type of local structure favors a random (amorphous) distribution of barium oleate molecules at the nanoparticle (nanocube) surface, and therefore cannot provide a lattice mismatch between the shell and the core. The complete assignment of IR absorption lines of the samples is in Table 1.^28–32^

**Table 1:** Spectroscopic assignments of IR absorption bands of ball-milled BaTiO_3_/barium oleate nanoparticles/nanocubes^28–32^

Frequency, cm^−1^	Intensity	Assignment
495	Weak	Ba–O, (TiO_3_)
720	Weak	CH_2_ rocking
930	Weak	*ω*_3_ COO^−^ deformation
1059	Weak	C–C stretching
1095	Weak	
1113	Weak	CH_3_ rocking
1240	Weak	
1279	Weak	CH_2_ twisting, wagging
1320	Weak	
1344	Weak	
1408	Strong	*ω*_1_ COO^−^ symmetrical stretching
1433	Strong	
1466	Medium	CH_2_ bend {*δ* (CH_2_) scissoring}
1510	Strong	*ω*_1_ COO^−^ asymmetrical stretching
1540–1545	Strong	
2852	Strong	CH_2_ symmetrical stretching
2922	Strong	CH_2_ asymmetrical stretching

**Fig. 4:**
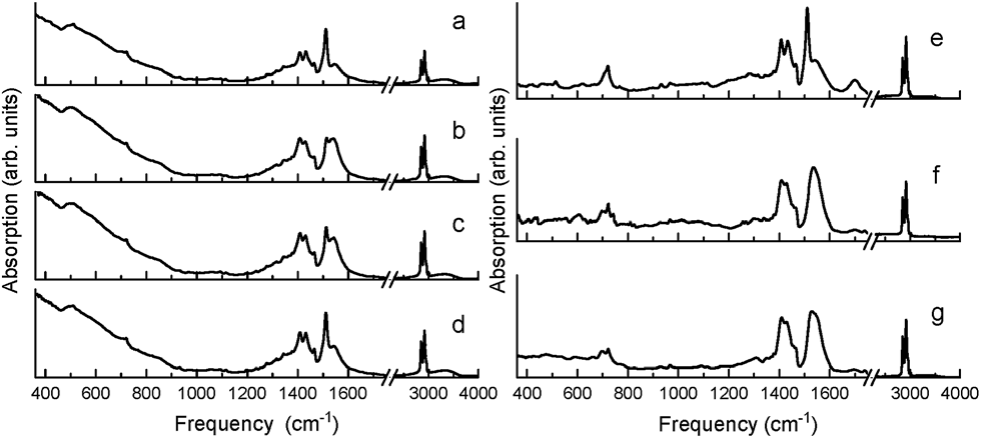
(Left) FTIR spectra of ball-milled BaTiO_3_ nanoparticles at room temperature. (a) Before annealing and (b–d) after annealing at 140 °C and cooling back to room temperature. The time between cooling to room temperature and the subsequent measurements varied: (b) 10 min, (c) 60 min, and (d) 300 min. (Right) FTIR spectra of milled commercial barium oleate at room temperature. (e) Before annealing, and (f and g) after annealing at 140 °C and cooling back to room temperature. The time between cooling to room temperature and the subsequent measurements varied: (f) 10 min and (g) 300 min. The spectra in (b) and (f) were measured 10 min after cooling to room temperature to assure for a uniform temperature throughout the sample.

Similar in nature to the transformation of the quasi-bridging to chelate coordination binding modes resulting from mechanochemical synthesis (*i.e.* high energy ball-milling), a transformation of the crystalline chelate bidentate to the amorphous quasi-bridging bidentate was observed by annealing the milled BaTiO_3_ nanoparticles (milled Aldrich powder from Fig. 1b) at 140 °C, which is above the Curie temperature. Fig. 4a–d show the FTIR absorption spectra of the ball-milled nanoparticles before annealing, and as a function of time at room temperature after cooling. It can be seen that annealing the sample drastically modifies the absorption spectrum, as shown in Fig. 4b (note, the spectra in Fig. 4b and f were measured 10 min after cooling the sample to room temperature to provide a uniform temperature throughout the sample). In particular, the intensity of the 1540 cm^−1^ band increased at the expense of the 1510 cm^−1^ band, *i.e.* amorphous metal carboxylates increased at the expense of crystalline metal carboxylates, which was proven to be a reversible process in Fig. 4 as the sample with a time delay on the order of hours (Fig. 4d) showed nearly a full recovery of the 1510 cm^−1^ band (*i.e.* a reversal of the amorphous quasi-bridging structure back to the crystalline chelate structure). This reconfigurable “self-healing” mechanism was observed only for samples that initially had the chelate structure. Unlike the reversible-nature of the oleate-shell surrounding the ball-milled nanoparticles, the annealing of milled commercial barium oleate resulted in a permanent transformation of the sample's bond structure to a quasi-bridging coordination binding mode with a broadening of asymmetrical and symmetrical absorption bands, shown in Fig. 4e–g.

A key observation is the similarity between Fig. 1c and 4b, where the pre-milled nanocubes (with the type I amorphous shell) have an absorption spectrum strongly resembling that of the annealed ball-milled nanoparticles (measured within minutes after cooling to room temperature). In both of these cases, the quasi-bridging coordination binding mode is dominant (note the lack of the 1510 cm^−1^ absorption band). The balled-milled nanoparticles measured hours after cooling (Fig. 4d) and the nanocubes measured after ball-milling (Fig. 1d) show an interesting conversion of the quasi-bridging coordination binding mode (type I, amorphous shell) to the chelating coordination binding mode (type II, crystalline shell). The milling of the nanocubes introduced a surface stress, by creating a crystalline oleate shell with a chelate coordination binding mode, which was removed *via* annealing resulting in a transformation to a quasi-bridging coordination binding mode (Fig. 4); over time (hours) the stress/strain was reapplied as the oleate shell transformed back to the ordered chelate structure.

The condition that would allow for such a stress/strain is manifested in the nature of the type II crystalline bonding structure and the lattice structure of the BaTiO_3_ core, where an epitaxial-like strain (mismatch between the shell and the core particle) is responsible for enhancing the ferroelectric properties of the nano-system, *i.e.* the superparticle. With the type II bond structure, a lattice mismatch exists between the crystalline shell and the core, which cannot exist with the type I amorphous shell. This is analogous to the lattice mismatch found in 2-D thin films.^20^

In order to compare the ferroelectric properties of BaTiO_3_ with either a type I shell (quasi-bridging, random distribution) or a type II shell (chelating, ordered arrangement), the pre- and post-milled nanocubes are ideal samples to use as their size and shape of the core BaTiO_3_ particle are practically the same (see Fig. 1 TEM images); therefore, any difference in ferroelectricity would certainly result from the carboxylate coordination binding mode. The AC displacement current density measurements (described in ref. 2, 3 and 10) of BaTiO_3_ nanocubes created by solvothermal synthesis reveal a remarkable 650 times difference in magnitude of the spontaneous polarization for pre- and post-ball-milled nanocubes, *P*_s_ = 0.2 and *P*_s_ = 130 μC cm^−2^, respectively. By way of comparison, the spontaneous polarization of ball-milled synthesized nanocubes is 5 times greater than that of bulk BaTiO_3_.^2^ These results are very different from the cases reviewed in ref. 33. The only difference between the pre- and post-ball-milled nanocubes is the shell carboxylate coordination binding mode; this is a strong indicator that the surface strain is the mechanism behind the enhanced spontaneous polarization, and the source of the strain is indeed related to an lattice mismatch between the crystalline organic shell and core nanoparticle. Note, the milled barium oleate alone does not contribute to the enhancement of the spontaneous polarization.

## 5. Conclusions

In summary, we report on a stabilization and enhancement of spontaneous polarization in 10 nm ball-milled BaTiO_3_ nanoparticles and nanocubes, which is linked to an *in situ* mechanochemically produced barium oleate with a crystalline chelate structure. This results in ferroelectric “hybrid” nanoparticles with an organic functional crystalline shell surrounding an inorganic core. The crystalline nature of the shell has been independently demonstrated in the results of both FTIR absorption and XRD measurements. Because of the synergistic effect of the core and the shell, this nanoparticle is described as a *superparticle*, where the spontaneous polarization/dipole moment belongs to the *system* (BaTiO_3_ inorganic core and barium oleate organic shell) and not just the nanoparticle itself.^2^ The mystery of the source/mechanism providing the surface stress predicted in ref. 11 has now been identified as an epitaxial-like strain between the inorganic core and the organic functional shell of the superparticle. The chelate structured carboxylate (type II shell) surrounding the core BaTiO_3_ nanoparticle is the primary source of the “epitaxial” strain/stress resulting from its crystalline nature (*i.e.* lattice mismatch), and is not achievable using conventional wet chemistry growth techniques. Temperature dependence of the IR absorption demonstrates the effects removing the surface stress *via* annealing, resulting in a transformation of the chelate coordination binding mode (crystalline) to a quasi-bridging coordination binding mode (amorphous). This process is shown to be reversible with a slow transformation back to its original chelate state exhibiting a self-healing effect.

The ordered chelate carboxylate shell is responsible for a 5 times enhancement of the spontaneous polarization with respect to BaTiO_3_ bulk crystal, and a 650 times enhancement of spontaneous polarization compared to synthesized (non-milled, type I amorphous shell) unstressed BaTiO_3_ nanocubes. Thus, ball-milled 10 nm BaTiO_3_ nanocubes surrounded with such (type II) ligands exhibit excellent ferroelectric properties, which are relatively homogenous in shape and size. The ability to create ferroelectric nanoparticles with both a large spontaneous polarization and a uniform shape may further advance numerous fields, for example, in liquid crystal applications they would provide a means for increasing the field sensitivity, while not introducing a plethora of defects from randomly shaped particles that could modify liquid crystal parameters such as phase behavior, transition temperatures, and long-range ordering.^34^

## Conflicts of interest

The authors declare no competing financial interests.

## Supplementary Material
